# Expression of Innate Immunity Genes and Damage of Primary Human Pancreatic Islets by Epidemic Strains of Echovirus: Implication for Post-Virus Islet Autoimmunity

**DOI:** 10.1371/journal.pone.0077850

**Published:** 2013-11-01

**Authors:** Luis Sarmiento, Gun Frisk, Mahesh Anagandula, Eduardo Cabrera-Rode, Merja Roivainen, Corrado M. Cilio

**Affiliations:** 1 Cellular Autoimmunity Unit, Department of Clinical Sciences, Skåne University Hospital, Lund University, Malmo, Sweden; 2 Department of Virology, “Pedro Kouri” Tropical Medicine Institute, Havana, Cuba; 3 Department of Immunology, Genetics and Pathology, Uppsala University, Rudbeck Laboratory, Uppsala, Sweden; 4 Department of Immunology and Genetics on Diabetes, National Institute of Endocrinology, Havana, Cuba; 5 Intestinal Viruses Unit, Division of Health Protection, Department of Infectious Disease Surveillance and Control, National Institute for Health and Welfare (THL), Helsinki, Finland; La Jolla Institute for Allergy and Immunology, United States of America

## Abstract

Three large-scale Echovirus (E) epidemics (E4,E16,E30), each differently associated to the acute development of diabetes related autoantibodies, have been documented in Cuba. The prevalence of islet cell autoantibodies was moderate during the E4 epidemic but high in the E16 and E30 epidemic. The aim of this study was to evaluate the effect of epidemic strains of echovirus on beta-cell lysis, beta-cell function and innate immunity gene expression in primary human pancreatic islets. Human islets from non-diabetic donors (n = 7) were infected with the virus strains E4, E16 and E30, all isolated from patients with aseptic meningitis who seroconverted to islet cell antibody positivity. Viral replication, degree of cytolysis, insulin release in response to high glucose as well as mRNA expression of innate immunity genes (IFN-b, RANTES, RIG-I, MDA5, TLR3 and OAS) were measured. The strains of E16 and E30 did replicate well in all islets examined, resulting in marked cytotoxic effects. E4 did not cause any effects on cell lysis, however it was able to replicate in 2 out of 7 islet donors. Beta-cell function was hampered in all infected islets (P<0.05); however the effect of E16 and E30 on insulin secretion appeared to be higher than the strain of E4. TLR3 and IFN-beta mRNA expression increased significantly following infection with E16 and E30 (P<0.033 and P<0.039 respectively). In contrast, the expression of none of the innate immunity genes studied was altered in E4-infected islets. These findings suggest that the extent of the epidemic-associated islet autoimmunity may depend on the ability of the viral strains to damage islet cells and induce pro-inflammatory innate immune responses within the infected islets.

## Introduction

Type 1 diabetes results from the autoimmune destruction of insulin-producing beta cells. Genetic and epidemiological evidence points to an overriding environmental influence on type 1 diabetes development. Among examined environmental agents, human enteroviruses (HEV) appear to play a prominent role [1].

HEV are antigenically variable infectious agents of the Picornaviridae family, that includes over 100 different virus types, grouped into species A (17 Serotype), B (58 serotype), C (20 serotype) and D (4 serotype) [2]. Accumulating evidences indicates that diabetogenicity seems to be associated mainly to the B HEV species especially the group B coxsackieviruses (CVB1-6), although it is not restricted to one particular strain [3]. While the CVB serotypes are often described as viral triggers of type 1 diabetes, some Echoviruses (E) have also been associated with type 1 diabetes, at the time of clinical presentation or at preclinical stages [4]–[8].

One noteworthy and striking observation is that the global distribution of new cases of type 1 diabetes is not homogeneous, with the highest incidence rate found in Nordic countries and the lowest in tropical and subtropical regions [9]. Thus, it is somehow paradoxical to propose HEV as diabetogenic, particularly in the tropics where exposure to these agents is common and occurs year-round with possible peaks of enteroviral diseases as aseptic meningitis in the summer-fall season [10].

In Cuba, large nationwide epidemics of meningitis due to echovirus type 4, type 16, and type 30 have been documented in 1986, 2000, and 2001, respectively [10], [11]. One of the most notable observations from these HEV epidemics is that in the convalescent, but not in the acute stage of the infection, islet cell antibodies (ICA) have emerged. The islet cell autoantibodies was clearly infection-associated, since no serum samples from uninfected subjects serologically negative for neutralizing antibodies to E4, E16 and E30 had ICA [12]–[14].

Interestingly, infection-associated islet autoantibodies showed subtle differences among these epidemics in terms of prevalence and antigen specificity. The prevalence of ICA during the 2000 and 2001 meningitis epidemic was as high as 92.1% (35/38) and 87.5% (7/8), respectively; however, ICA prevalence was moderate (36.1%, 48/133) in the 1986 epidemic [12]–[14]. Remarkably, in the epidemic caused by E16 and E30 the emergence of insulin autoantibodies (IAA) and glutamic acid decarboxylase autoantibodies (GADA) was detected [8]. Another interesting observation from E16 epidemic in Cuba is that the titer of islet-associated autoantibodies correlates with the virus-neutralizing antibody titers. This suggests that the extent of infection and the antiviral immune response could influence the intensity of the humoral response against host antigens [8]. However, the mechanisms through which HEV influence development of islet autoimmunity are as yet unknown.

Current evidences suggest that post-viral islet autoimmunity will depend upon a combination of two qualities; the ability of a virus to cause beta cell damage and the ability to induce pro-inflammatory innate immune activation [15], [16]. Indeed, several groups have published data which indicate that HEV may target islet cells. The CV-B4 E2 diabetogenic strain and the CV-B4 VD2921 strain as well as the prototypes of CV-B2, 3, 4 and 5 serotypes, can infect and damage human beta-cells in vitro [17]–[21]. Other researchers demonstrated that various HEV strains, frequently encountered in the population and in the environment from countries with a high type 1 diabetes incidence, can replicate in cultured human islets with beta-cells infection and islets destruction [22]. On the other hand, it has been proposed that during viral infection the local production of cytokines and chemokines by both invading immune cells and the pancreatic beta cells themselves may interact to initiate and/or accelerate the transition from innate immune response to a chronic autoimmune assault [23]–[25].

In this study, echovirus isolates from three meningitis epidemic in Cuba, each associated to a varying degree with the acute development of islet cell autoantibodies, were used. The aim was to determine the effects of such an epidemic strains on beta-cell lysis, beta-cell function and innate immunity gene expression in human pancreatic islets. In addition, a possible genetic relationship between E30 isolates from 2001 Cuban meningitis epidemic and E30 isolates established previously as highly islet cell destructive strains was studied by phylogenetic analysis.

## Materials and Methods

### Tissue Source and Ethics Statement

Human pancreases were obtained from seven organ donors free of any known pancreatic disease, from transplantation units in Sweden, Norway, and Finland. Written ethical approval was provide to Prof. Olle Korsgren, Dept. of Radiology, Oncology and Clinical Immunology, Uppsala University Hospital by the regional ethics committee at the t, ref. no. DNR 2009/043. In all cases informed written consent was obtained from all donors or donor relatives. Islets of Langerhans were isolated in Uppsala, Sweden, using a protocol approved by the local ethics committee, as described previously [26]. Isolated human islets had a purity ranging from 40% to 95%, and available for research only because the total islet volume was too low for clinical transplantation. See table S1 for donor information.

### Viruses

Clinical strains of E4, E16 and E30 isolated during the Cuban meningitis epidemic in the years 1986, 2000, and 2001, respectively were included in the study [12]–[14]. All three strains were stool isolate from patients with aseptic meningitis that developed ICA, during the convalescent but not during the acute stage of the infection. In addition, the meningitis patient infected with E16 developed autoantibodies against Tyrosine phosphatase (IA2A), GADA, and IAA while the patient infected with E30 developed GADA and IA2A. The patient with the echovirus 4 infection developed ICA, but not GADA, IAA or IA2A [12]–[14]. ICA was assayed in all three cases by using a similar workshop - validated immunofluorescence assay. Biochemical type 1 diabetes-associated autoantibodies were measured using commercially available immunoassay kits validated in similar workshop [12]–[14]. For viral isolation, 200 µl of fecal specimens were inoculated in duplicate into tubes covered with monolayers of Green Monkey Kidney (GMK) cells. The inoculated cultures were subpassaged weekly at least 2 times in order to obtain the typical degenerative Cytopathic Effect (CPE) of enterovirus (e.g., cell rounding followed by the shrinkage and degeneration of the cell sheet). The identity of all isolates used was confirmed by neutralization tests with type-specific antisera and partial VP1 sequences by the use of primer pairs 011 and 187 as described by Oberste et al [27].

### Infection of Isolated Islet

The islets were kept in culture bags (Baxter Medical AB, Kista, Sweden) with 200 ml CMRL-1066 (ICN Biomedicals, Costa Mesa, CA) supplemented with 10 mM HEPES, 2 mML-glutamine, 50 mg/ml Gentamycin, 0.25 mg/ml Fungizone (Gibco BRL, Invitrogen Ltd, Paisley, UK), 20 mg/ml Ciproxfloxacin (Bayer Healthcare AG, Leverkusen, Germany), 10 mM nicotinamide, and 10% heat-inactivated human serum at 37°C in 5% CO_2_ and humidified air for 1–7 days. To further increase islet purity, human islets were handpicked with a micropipette under an inverted light microscope. All experiments were performed on 50 hand-picked islets per well, and cultured in non-attach six-well plates (Sarstedt, Nümbrecht, Germany) in 2 mL RPMI containing 5.5 mM glucose (SVA, Uppsala, Sweden), supplemented with 10% foetal bovine serum and 2 mM L-glutamine. Free floating islets were infected with a 1000 cell culture infectious dose-50 (CCID50)/0.1 mL of each viral strain. Two wells containing islets from the same donor were not infected and used as a control.

### Cytopathic Effects and Virus Titrations

The cytolytic effect of the clinical echovirus strains on the primary human pancreatic islets was studied for a period of 3–5 days, depending of the appearance of cytopathic effect (CPE)/islet dissociation or not. The islets were examined each day in a light microscope. The degree of CPE was graded from 0 to 4+, with 0 indicating no CPE when compared to the uninfected control and 4+ being a total destruction of the islets. Virus titers in the culture medium from infected islets were determined by the CCID50 titration method on GMK cells. Briefly, 10-fold dilutions (1∶10 to 1∶10^6^) of samples of the culture medium which had been collected on day 0, day 3 or day 5 post-infection (dpi), were added in triplicate to GMK cells cultured in 96-well plates. The CPE was read on day 5, and CCID50 titer was calculated using the Karber formula [28]. The extent of virus replication was expressed as the difference between the CCID50 titres at 3 or 5 dpi and at 0 dpi (samples of culture medium collected directly after infection).

### Immunocytochemistry (IHC)

For IHC infected and uninfected islets were washed in phosphate-buffered saline (PBS), fixed in 4% buffered PFA, dehydrated in graded ethanol and embedded in paraffin. The paraffin embedded islets were sectioned (5 µm) and dried on Superfrost glass slides (Menzel-Gläzer, Fischer scientific, Braunschweig, Germany), followed by de-paraffinization and re-hydration in 99-70% Ethanol. Antigen retrieval was performed in pH 9 TE-buffer (DAKO, Glostrup, Denmark) in a steam boiler and permeabilized in PBS containing 0.05% TWEEN 20. Endogenous peroxidase was blocked by the use of a ready-to use peroxidase blocker (DAKO, Glostrup, Denmark). After rinsing with PBS for 10 min, they were stained at room temperature with monoclonal antibody directed a broad reacting epitope on the structural EV Protein 1 (VP1), monoclonal antibody which specifically recognize double-stranded RNA and insulin-specific monoclonal antibody. Incubation with these antibodies was performed at room temperature for one hour and the visualization was achieved by the anti-mouse Envision-kit (Dako, Glostrup, Denmark) using DAB as substrate chromogen.

### Beta Cell Function Test

Insulin secretion in response to glucose stimulation was assessed in a dynamic perifusion system on day 3 of culture in the culture medium. Islets were perifused with two glucose concentrations (1.67 and 16.7 mmol/L and then 1.67 mmol/L again). Fractions were collected at 6-min intervals for 120 min and the insulin concentrations were determined by ELISA (Mercodia, Uppsala, Sweden).

### Innate Immunity Gene Expression in Echovirus-infected Islets

Three days post infection isolated human pancreatic islets were washed in PBS and lysed by using RLT buffer (Qiagen, Sollentuna, Sweden) on QIAshredder spin columns (Qiagen, Sollentuna, Sweden). Total RNA from islets was extracted with RNAeasy Mini kit with on-column DNaseI digestion (Qiagen) with G DNA eliminator column to remove the genomic DNA. RNA quantity and quality were determined with Nanodrop (Thermo Scientific, Braunschwig, Germany). Fifty nanograms of total RNA per sample were primed with random hexamer and reverse transcribed to cDNA with SuperScript IITM RT (Invitrogen) according to the manufacturer’s instructions. Control reactions containing all reagents except the reverse transcriptase were included to confirm the absence of interfering genomic DNA. RNase OUT™ (Invitrogen) was added to the reaction to avoid RNA degradation. Reaction was carried out at 250C for 10 min, 42°C for 55 min and 15 min at 75°C.

Real-time PCRs were run with SYBER Green master mix (Applied Biosystems, Stockholm, Sweden) in a 96 well optical plate on a StepOnePlusTM Real-Time PCR system (Applied Biosystems, Stockholm, Sweden). The cycling conditions were 40 cycles of 15 sec at 94°C, 30 sec 55°C and 30 sec at 68°C. Predesigned Genes specific primer sets (QuantiTect1 Primer Assays, Qiagen) were used for detection of regulated activation normal T expressed secreted (RANTES), toll-like receptor 3 (TLR3), melanoma-differentiation-associated gene 5 (MDA5), retinoic-acid-inducible protein I (RIG-I), 2′–5′-oligoadenylate synthetase 1 OAS, and Interferon beta (IFN-b) cDNA. Real time PCR data was analyzed by comparative delta ct method. The expression level of each gene was normalized to the expression of the 18 s house keeping gene by subtracting the 18 s ct value from each gene ct values. The relative gene expression levels were calculated by using 2∧-dct formula. Melt curve analysis was used to verify the specificity of final PCR products.

### E30 Nucleotide Sequence and Phylogenetic Analysis

The strain of E30 associated with islet cell autoantibodies used to infect the human islets in the present study as well as another nine isolates of E30, representing a cross-section obtained at the beginning, mid-stage and end of the 2001 epidemic in Cuba were included in the sequence analysis. Total RNA of ten isolates of E30 was extracted from culture supernatant from infected cells using QIAmp viral RNA Mini kit according to the manufacturer’s instructions (QIAGEN GmbH, Hilden, Germany). cDNA was synthesized from purified RNA by use of random hexamer primers and a ThermoScript preamplification kit (Life Technologies, Inc.). The VP1 region of each isolate was amplified as a single fragment by using specific primer pairs 011/187 (Oberste et al., 1999). PCR products were gel isolated and purified for sequencing with the QIAquick Gel Extraction kit (QIAGEN, Inc.). Cycle sequencing reactions were performed using the same forward and reverse primers, as in the PCR. Both forward and reverse reactions were performed to resolve possible ambiguities. An automated DNA sequencer was used for sequencing the VP1 fragment. Sequence analysis was performed using the Molecular Evolutionary Genetics Analysis Version 4.0 (MEGA 4) and Geneious v5.5 [29]. The phylogenetic tree was constructed by means the neighbor-joining method with bootstrapping of 1000 replicates. The evolutionary distances were computed using the Maximum Composite Likelihood method. All positions containing alignment gaps and missing data were eliminated only in pairwise sequence comparisons (Pairwise deletion option). The E30 sequences were compared to those of the GenBank sequence database and to those previously reported as highly islet cell destructive [30].The nucleotide sequence reported in this study has been submitted to GenBank database under the following accession numbers: KC 407676 to KC 407685.

### Statistical Analysis

All results were based on observations from at least three donors. Data are presented as means± SD. Statistical analysis was performed using SPSS package for Windows, version 12.0.1. Differences in insulin release and expression of innate immunity genes between the three isolates and uninfected controls were analyzed by Kruskal-Wallis test. Comparisons between each isolate and uninfected control were performed using the Mann-Whitney *U* test. P-values lower than 0.05 were considered statistically significant.

## Results

### Islet Cell Cytolytic Capacity of Epidemic Echovirus Strains

**Figure 1:**
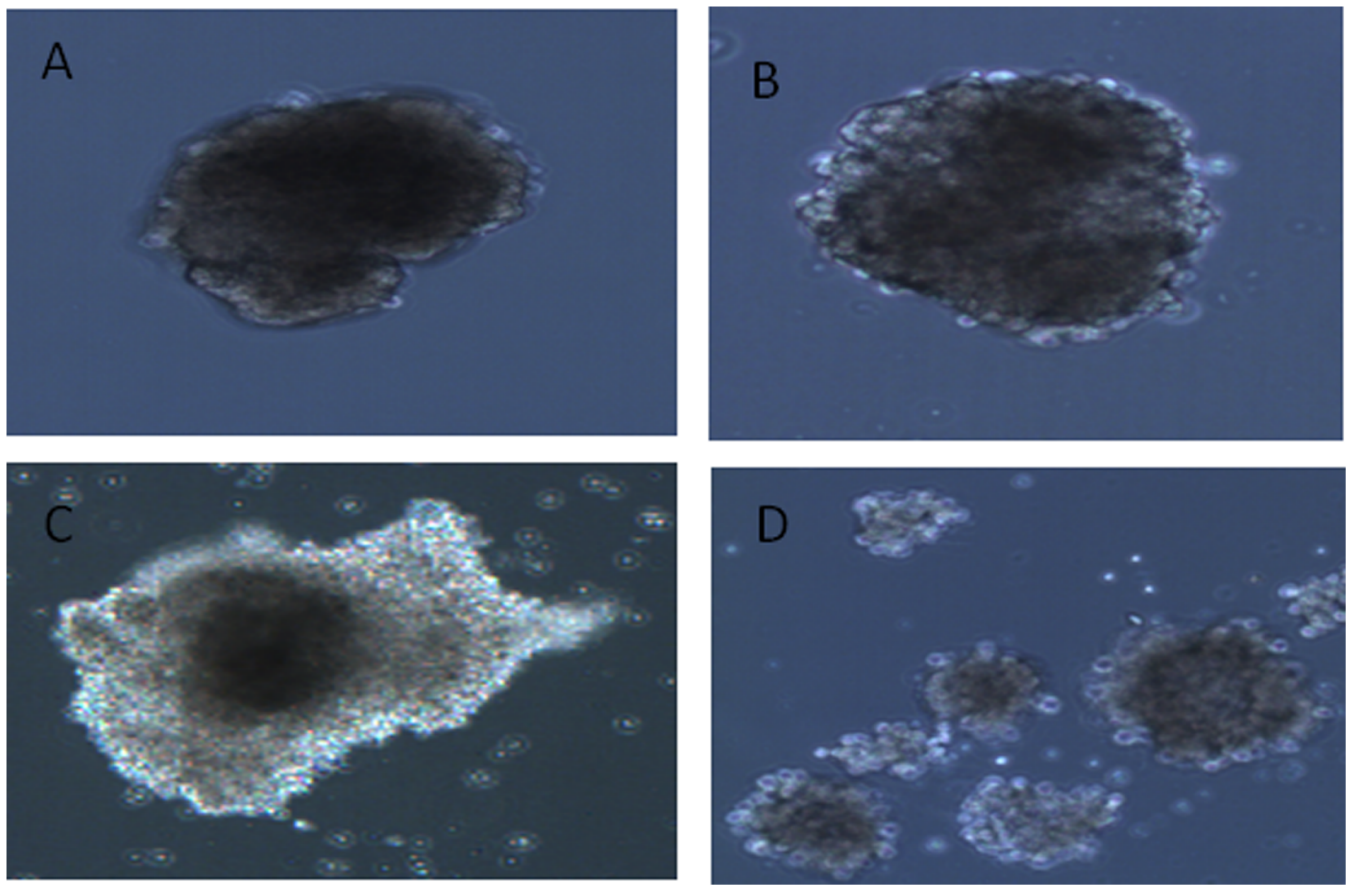
Virus-induced cytopathic effect in primary human pancreatic islets cells. A. Uninfected islet. B. Islets infected with the E4 isolate 5 days post infection. C. Islets infected with E16 isolates 3 days post infection. D. Islets infected with E30 isolates 3 days post infection. The figure is representative of seven islet donors.

**Figure 2:**
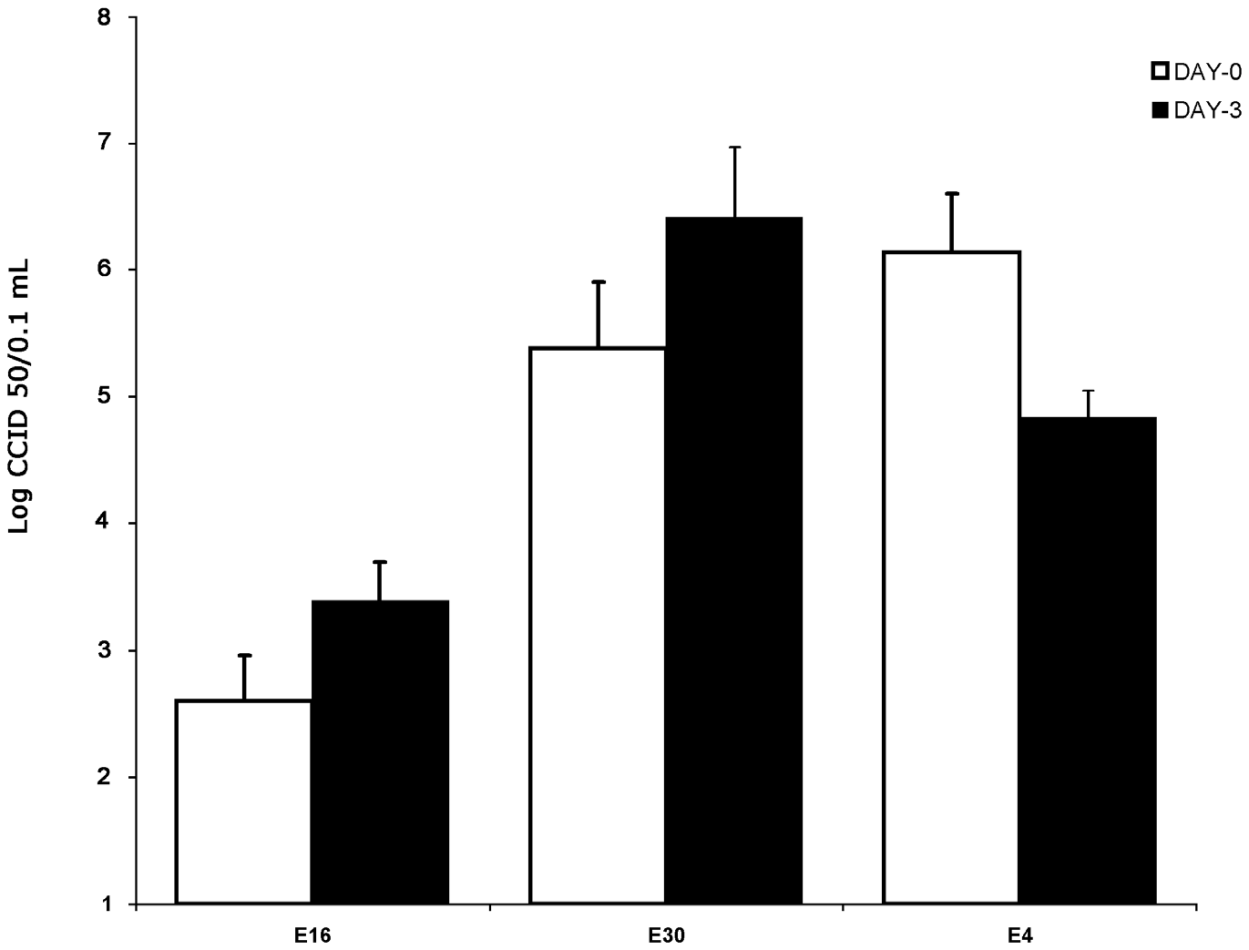
Viral titers of the clinical strains of E16, E30 strains and E4 in the culture medium of infected primary human islets during 3 days post-infection. Aliquots of the culture medium were withdrawn day 0 and day 3. Virus titers were obtained using the cell culture infectious dose 50 (CCID50) titration methods. The results are shown as the means ± SD from experiments performed in triplicate.

Clinical strains of E16 and E30 caused destruction of the isolated human pancreatic islet from seven different donors. The first morphological changes were seen as early as one day after infection in all islet infected with E16 and E30. At three days after infection the virus-induced cytopathic effect became more pronounced. Islet degeneration was characterized by the loss of islet integrity, disintegration, and partial dispersion of islets. There was no difference in degree of islet destruction between islets infected with E16 or E30 (Fig. 1). Islets infected with E4 did not show any virus-induced effect at any time post infection (Fig. 1). Isolates of E16 and E30 did replicate well in islet cell with a titer rise from day 0 to day 3. The mean CCID50 titers increase in islet infected with strains of E16 and E30 was 0.7 logCCID50 and 1.2 logCCID50, respectively. The CCID50 titers were decreased (1.3 logCCID50) in E4-infected islets from 5 donors (Fig. 2). Strikingly, the CCID50 titers increased (1 logCCID50) in 2 out of 7 (28.5%) donor islets infected with E4 between day 3 and 5 post infection (not shown). However, no CPE could be seen in islets from any of the seven donors suggesting there was replication in islets from two donors without inducing CPE.

Islets infected with E16 and E30 revealed positive immunostaining when using insulin, EV protein 1 and double-stranded RNA specific monoclonal antibody indicating that these virus strains infected and replicated in insulin containing cells. Islets inoculated with the E4 strain were only stained positive with the insulin specific antibody.

### Insulin Release in Response to High Glucose Stimulation in Echovirus-infected Islets

**Figure 3:**
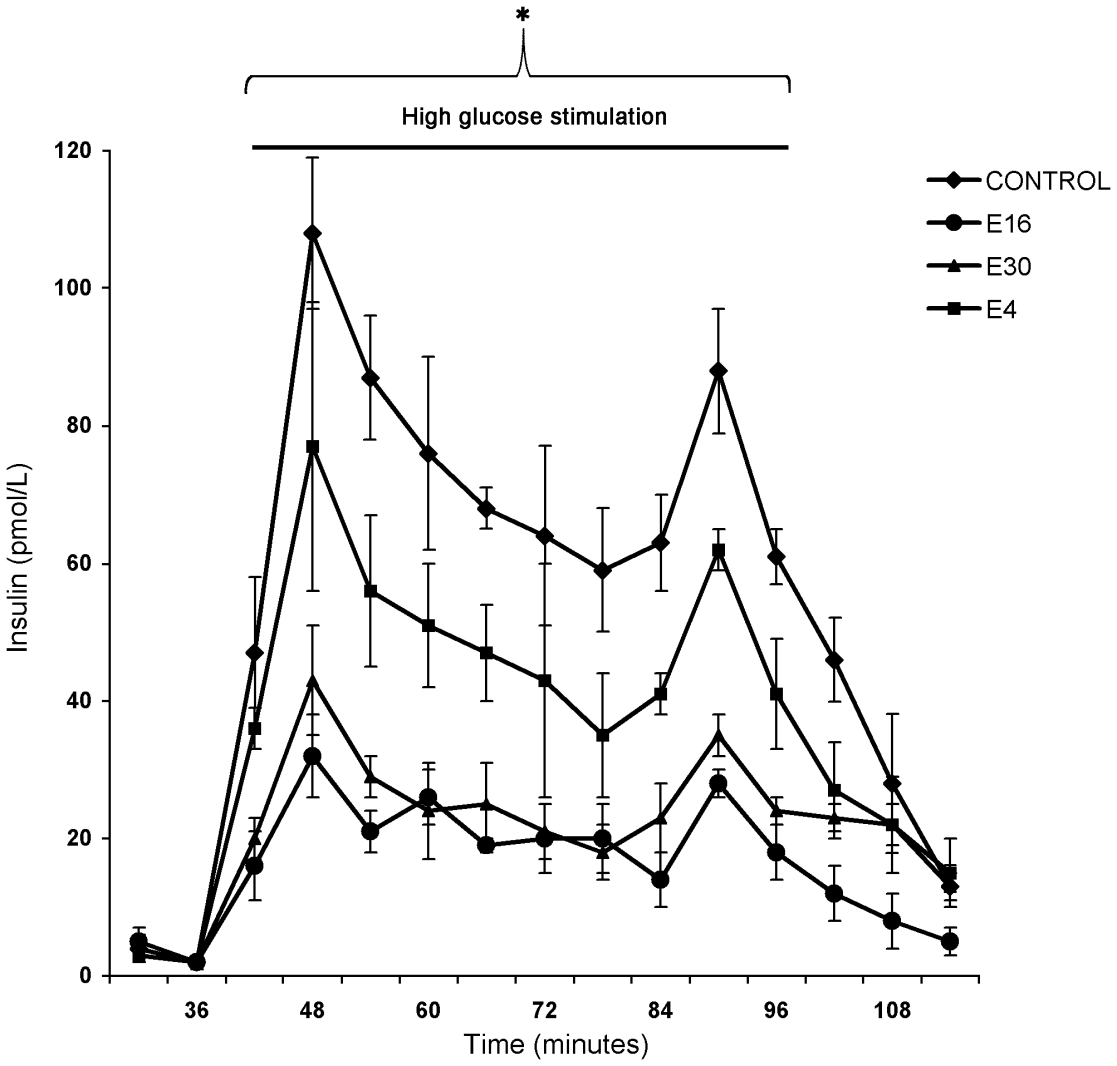
Dynamic release of insulin in primary human islet cells after perifusion with glucose (1.67, 16.7, and 1.67 mmol/L) at three days after infections with clinical strains of E4, E16 and E30. Data are presented as means ± SD and were based on observations from at least three donors. *P<0.05 between groups (minutes 42 to 102); Kruskal-Wallis test.

The ability to secret insulin in response to high glucose was reduced significantly in islets inoculated with E4, E16 and E30 when compared to uninfected islet (p<0.05). Despite that beta-cell function was hampered in all infected islets, the effect of the epidemic strains of E16 and E30 on insulin secretion appeared to be higher than the epidemic strain of E4 (Fig. 3).

### Effect of Epidemic Echovirus Strains on Expression of Innate Immunity Genes by Human Pancreatic Islets

**Figure 4:**
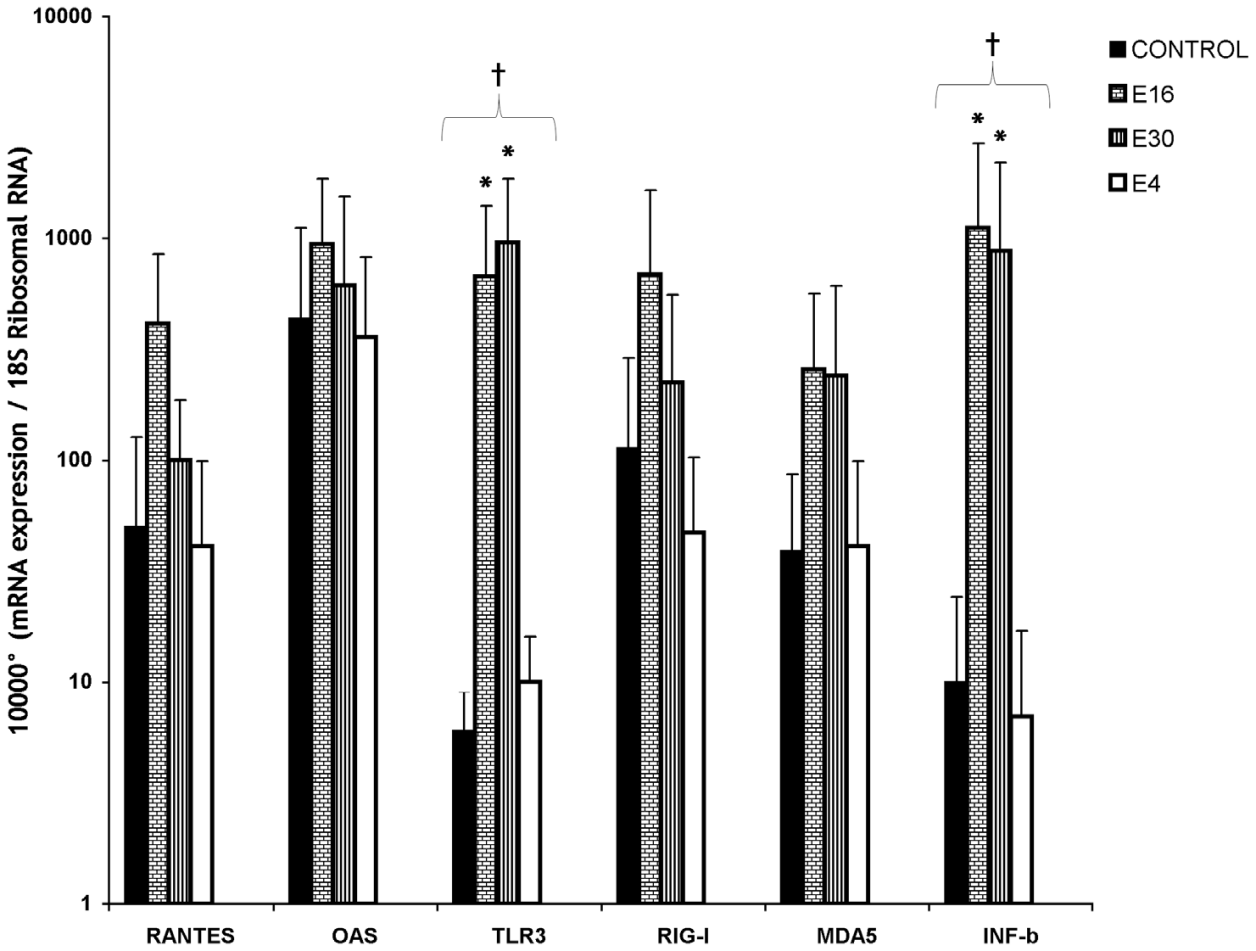
Innate immunity gene expression in primary human islets cultured after three days of infection with clinical strains of E4, E16 and E30. Gene expression levels are presented as mRNA expression relative to expression of the housekeeping gene 18(2^_^dct). Data are presented as means ± SD and were based on observations from at least three donors. ^†^ *P*<0.05, between all groups (Islets infected with E4, E16, E30 and uninfected controls). *P<0.05, Islets infected with E16 and E30 were compared to E4-inoculated islets and the uninfected control.

Since recent studies reported that *in vitro* infection of human islets with HEV led to increased transcription of genes encoding IFN- b TLR 3, MDA5, RIG-I, RANTES and OAS [31], [32], we examine the expression of these innate immunity genes in isolated human pancreatic islets after infection with epidemic strains of echovirus. TLR3 and IFN-b mRNA expression increased significantly following infection with E16 and E30 (P<0.033 and P<0.039 respectively) compared to E4-inoculated islets and the uninfected control (Fig. 4). Infection with E16 and E30 also led to increased transcription of the innate immunity genes RIG-I, RANTES and OAS although this did not reach statistical significance (Fig. 4). In contrast, the gene-expression of RIG-I, TLR3, RANTES, OAS, and IFN-b, were not altered in E4-infected islets (Fig. 4).

### Phylogenetic Clustering of E30 Isolates

**Figure 5:**
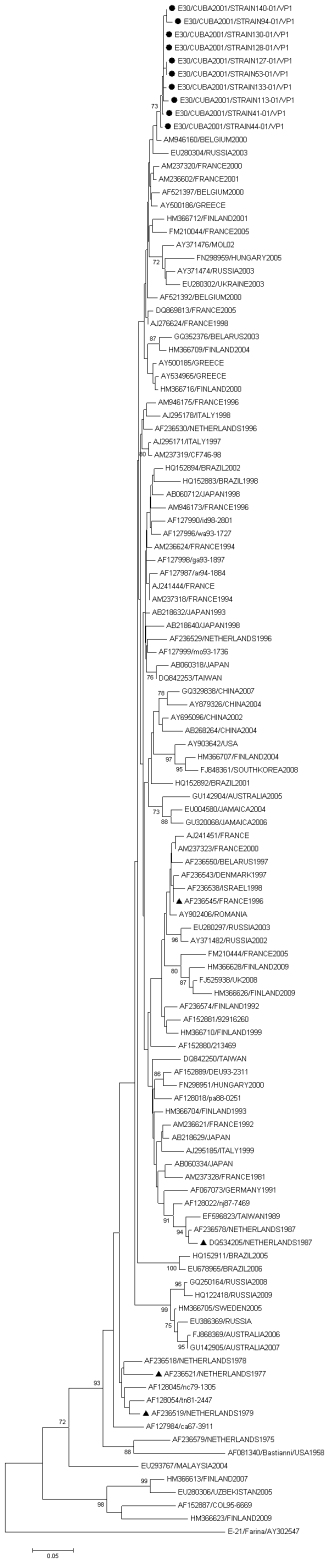
Dendrograms showing phylogenetic relationships between sequenced E30 isolated during the Cuban epidemic of aseptic meningitis in 2001 and the E30 isolate of the GenBank sequence database. The percentage of replicate trees in which the associated taxa clustered together in the bootstrap test (1000 replicates) is shown next to the branches. Strains of E30 known to be highly destructive of primary human insulin producing beta cells are shown in the tree by small black triangles.

All E30 strains isolated during the Cuban epidemic of aseptic meningitis in 2001 are clustering together. Their close relatives were detected in different countries in years 2000–2005. The cluster identified in the study did not correlate with the genetic sub-clusters of isolates established previously as highly islet cell destructive strains (Fig. 5).

## Discussion

Since 1986, public health surveillance strategies in Cuba have identified three meningitis epidemics that were caused by echovirus type 4, 16, and 30, during each of which convalescent sera of cases with confirmed echoviral infection have shown seroconversion to ICA, the immunological hallmark of type 1 diabetes [12]–[14]. Interestingly, ICA prevalence was moderate during the E4 epidemic but high in the subsequent epidemics [8], [12]–[14]. Here we studied, for the first time, the effect of infection with epidemic strains of echovirus with proven but varying potency in their ability to induce islet cell autoantibodies on isolated human pancreatic islet.

The strains of E16 and E30 isolated from Cuban epidemics during the years 2000 and 2001, respectively did replicate well in all islets examined resulting in marked cytotoxic effects and impaired insulin secretory ability. Surprisingly, although the E4 strain isolated during the 1986 epidemic has no apparent cytolytic effects on islets, it was able to replicate in 2 out of 7 donor islets and caused functional damage of beta cells. However, the effect of the epidemic strains of E16 and E30 on glucose stimulated insulin secretion appeared to be higher than that of the epidemic strain of E4.

It is important to notice that in mice inoculated with the E4 strain circulating during the late ‘80 s in Cuba there was a reduction of the insulin concentration and overall protein synthesis in pancreatic islets [33]. At that time, Uriarte and colleagues also found that a significant proportion of the infected children developed temporary glucose intolerance [34]. Indeed, our results are further supported by the findings of Elshebani et al. [35], which found that insulin response was reduced in infected islets with HEV strains isolated from type 1 diabetic patients at clinical presentation of type 1 diabetes, even in the absence of direct cytolysis. Thus, it is in line with the view that HEV infection of islet cells could eventually lead to cellular stress resulting in the engulfment of the infected islet cells and presentation of normally sequestered islet antigens, a mechanism that has been referred to as bystander damage [36], [37].

Since there was replication of E4 strains in only 28.5% of the donors even in absence of CPE, we hypothesized that donor related variation in islet-cell susceptibility to E4 infections would explain the moderate prevalence of ICA during E4 epidemic. Factors influencing the donor-related differences in islet-cell susceptibility to infections are poorly understood. However, donor-specific factors, such as cytokine response capability, HLA-types and receptor usage could be important. Accordingly, it is tempting to speculate that only a subpopulation has islets that express receptors that this strain can bind to. This hypothesis need to be further elucidated with more detailed experiments.

The effects of the clinical echovirus strains on cell lysis and replication did correlate with the expression levels of innate immunity gene in primary human islet cells. While E16 and E30 lysed and replicated well in all infected islet cells, E4 did not, and increased expression of innate immunity genes by were only observed with the strains of E16 and E30. Previous studies have demonstrated that lytic enterovirus strains are more potent inducers of a pro-inflammatory micro-environment and probably recruit immune cells to the site of infections more efficiently than non cytolytic enterovirus strains do [38]–[40]. Indeed, our results fit well with the recent finding that the expression of proinflammatory cytokine genes (IL-1α, IL-1-β and TNF-α) that also mediate cytokine-induced beta cell dysfunction correlated with the lytic potential of a virus [40].

Activated innate immune responses including virus sensing by RIG-I, MDA5 and TLR3 with induction of OAS and other proinflammatory cytokines and chemokines expression such as IFN-b and RANTES will not only limit viral replication but will also enhance the adaptive immunity cascades for islet cell destruction [41], [42]. Thus, local induction of innate immunity response upon infection by echoviruses exhibiting islet tropism might be capable of providing a so called “fertile field” for islet autoimmunity.

Although the use of primary human islets isolated from their native environment poses limitations to the extrapolation of the finding obtained to the in vivo autoimmunity situation, the findings from this study suggest that the extent of the autoimmunity in human may depend on the ability of the viral strains to damage islet cells and to induce pro-inflammatory innate immune activation within the infected islet. Thus, it is highly plausible that the differential post-echovirus islet autoimmunity occurred during the meningitis epidemic in Cuba might be a result either of differences in the ability of the viruses to induce pro-inflammatory innate immune activation in islets or a differential islet cell killing capacity of the epidemic strains of echovirus.

Previous studies on human pancreatas or cultured islets have shown that there considerable variations in cytolytic and replicating effect of enterovirus on beta cells, not only between various viral serotypes, but also between strains of the same serotype [18], [20], [30]. Though not yet demonstrated, distinctive features in echovirus structure should be critical for beta-cell tropism. However, the highly destructive strains of E30 isolated in Cuba have found in separate clusters among the other less destructive ones. Likewise, the European E30 strains found to be highly islet cell destructive in previous studies are not clustering together but found in different subclusters [30]. Thus, the potentially diabetogenic E30 strains do not form phylogenetically distinct lineages, but cluster together with other circulating strains of respective types.

Even though the capsid coding region is the most variable part of the enterovirus genome and thus carrying the most robust phylogenetic signal connected to serotype specificity, there is increasing evidence that pathogenic properties of a viral strains strongly depend upon small sequence variations within a virus population (i.e. quasispecies biology) [40]. As suggested by studies by Al-Hello et al [43], it is possible that the most critical determinants for islet cell destruction might be hiding in their genomes or, outside of the structural protein coding region. Altogether these results support the previous findings [30], [40], [44] suggesting that small strain specific variations, rather than serotype identity, are essential for the tropism and thus diabetogenicity of HEV.

It is worth noting that our group is carrying out a prospective study in a great number of subjects who were infected by the E4, E16 and E30 (including the patients of the present study) to evaluate if there is any persistence of type 1 diabetes associated autoantibodies or progression toward overt type 1 diabetes. However, screening of the type 1 diabetes registers from the Cuban type 1 diabetes prediction program revealed that no subjects who were positive for ICA during the 1986, 2000 and 2001 echovirus meningitis epidemics developed type 1 diabetes from 1986 through 2012. In addition, no peak of type 1 diabetes incidence was reported during the 25-year period in Cuba (Sarmiento and Cabrera-Rode, unpublished observations).This suggests that virus-induced islet cell autoantibodies could be transient and may remit, and do not imply the clinical onset of type 1 diabetes. Otherwise, a wave of type 1 diabetes new cases should be expected after large echovirus epidemics in Cuba. On the other hand, type 1 diabetes incidence in Cuba is low (2.9×100,000 inhabitants) [45], [46], which means that persistence of islet cell autoantibodies generated during viral infection and clinical expression of diabetes are unlikely to occur in all individuals.

In line with the so called “hygiene hypothesis”, it is reasonable to assume that while the beta cell autoimmunity may be triggered upon exposure to HEV that target beta cells and promote inflammation within the islets, as indicated by studies of meningitis epidemic in Cuba, the host protective immune mechanism induced by protective HEV infections early in life could halt islet destruction and eventually abort the autoimmune process [47], [48]. Based on these view, further studies are needed to better understand how HEV can manipulate the host response to stimulate protection from type 1 diabetes, which will pave the way for designing novel preventive approaches.

## Supporting Information

Table S1 **Donor information.** DI, Dynamic index: n.a, not available.
(DOC)

## References

[1] Hyoty H, Taylor KW (2002) The role of viruses in human diabetes. Diabetologia 45: 1 353–1361. 10.1007/s00125-002-0852-3 12378375

[2] Oberste MS, Maher K, Nix WA, Michele SM, Uddin M, et al (2007) Molecular identification of 13 new enterovirus types, EV79–88, EV97, and EV100–101, members of the species Human Enterovirus B. Virus Res. 128: 34–42. 10.1016/j.virusres.2007.04.001 17485128

[3] Richer MJ, Horwitz MS (2009) The innate immune response: an important partner in shaping coxsackievirus-mediated autoimmunity. J Innate Immun 1: 421–434. 20375600 10.1159/000226247

[4] Otonkoski T, Roivainen M, Vaarala O, Dinesen B, Leipa JA, et al (2000) Neonatal Type I diabetes associated with maternal echovirus 6 infection: a case report. Diabetologia 43: 1235–1238. 11079741 10.1007/s001250051518

[5] Paananen A, Ylipaasto P, Rieder E, Hovi T, Galama JM, et al (2003) Molecular and biological analysis of echovirus 9 strain isolated from a diabetic child. J Med Virol 69: 529–537. 12601761 10.1002/jmv.10341

[6] Williams CH, Oikarinen S, Tauriainen S, Salminen K, Hyoty H, et al (2006) Molecular analysis of an echovirus 3 strain Isolated from an individual concurrently with appearance of islet cell and IA-2 autoantibodies. J Clin Microbiol 44: 441–448. 16455897 10.1128/JCM.44.2.441-448.2006 PMC1392672

[7] Al-Hello H, Paananen A, Eskelinen M, Ylipaasto P, Hovi T, et al (2008) An enterovirus strain isolated from a diabetic child belongs to a genetic subcluster of echovirus 11, but is also neutralized with monotypic antisera to coxsackievirus A9. J Gen Virol 89: 1949–1959. 18632967 10.1099/vir.0.83474-0

[8] Sarmiento L, Cubas-Dueñas I (2013) Cabrera-Rode (2013) Evidence of association between exposure to enterovirus and type 1 diabetes in Cuban children and adolescents. MEDICC Rev 15: 29–32. 10.37757/MR2013V15.N1.7 23396239

[9] Vehik K, Dabelea D (2011) The changing epidemiology of type 1 diabetes: why is it going through the roof? Diabetes Metab Res Rev 2: 3–13. 10.1002/dmrr.1141 21218503

[10] Sarmiento L (2004) Enteroviral meningitis and emergence of rare enterovirus types: Cuban experience. In: Strong Phyllis V (ed) Focus on meningitis research. Nova, New York, 1–14.

[11] Sarmiento L, Mas P, Goyenechea A, Palomera R, Morier L, et al (2001) First epidemic of echovirus 16 meningitis in Cuba. Emerg Infect Dis 7: 887–889. 11747705 10.3201/eid0705.017520 PMC2631890

[12] Uriarte A, Cabrera-Rode E, Ventura R, Vargas J (1987) Islet cell antibodies and Echo 4 virus infection. Diabetologia 30 (A): 590.

[13] Cabrera-Rode E, Sarmiento L, Tiberti C, Molina G, Barrios J, et al (2003) Type 1 diabetes islet associated antibodies in subjects infected by echovirus 16. Diabetologia 46: 1348–1353. 12898016 10.1007/s00125-003-1179-4

[14] Cabrera-Rode E, Sarmiento L, Molina G, Perez C, Arranz C, et al (2005) Islet cell related antibodies and type 1 diabetes associated with echovirus 30 epidemic: a case report. J Med Virol 76: 373–377. 15902705 10.1002/jmv.20368

[15] Richer MJ, Horwitz MS (2009) The innate immune response: an important partner in shaping coxsackievirus-mediated autoimmunity (2009) J Innate Immun. 1: 421–434. 10.1159/000226247 20375600

[16] Hober D, Sauter P (2010) Pathogenesis of type 1 diabetes mellitus: interplay between enterovirus and host. Nat Rev Endocrinol 6: 279–289. 20351698 10.1038/nrendo.2010.27

[17] Szopa TM, Ward T, Dronfield ND, Portwood ND, Taylor KW (1990) Coxsackie B4 viruses with the potential to damage b-cells of the islets are present in clinical isolates. Diabetologia 33: 325–328. 2165944 10.1007/BF00404634

[18] Roivainen M, Rasilainen S, Ylipaasto P, Nissinen R, Ustinov J, et al (2000) Mechanisms of coxsackievirus-induced damage to human pancreatic beta-cells. J Clin Endocrinol Metab 85: 432–440. 10634421 10.1210/jcem.85.1.6306

[19] Chehadeh W, Kerr-Conte J, Pattou F, Alm G, Lefebvre J, et al (2000) Persistent infection of human pancreatic islets by coxsackievirus B is associated with alpha interferon synthesis in beta cells. J Virol 74: 10153–10164. 11024144 10.1128/jvi.74.21.10153-10164.2000 PMC102054

[20] Frisk G, Diderholm H (2000) Tissue culture of isolated human pancreatic islets infected with different strains of coxsackievirus B4: assessment of virus replication and effects on islet morphology and insulin release. Int J Exp Diabetes Res 1: 165–175. 11467407 10.1155/EDR.2000.165 PMC2477734

[21] Ylipaasto P, Klingel K, Lindberg AM, Otonkoski T, Kandolf R, et al (2004) Enterovirus infection in human pancreatic islet cells, islet tropism in vivo and receptor involvement in cultured islet beta cells. Diabetologia 47: 225–239. 14727023 10.1007/s00125-003-1297-z

[22] Klemola P, Kaijalainen S, Ylipaasto P, Roivainen M (2008) Diabetogenic effects of the most prevalent enteroviruses in Finnish sewage. Ann NY Acad Sci 1150: 210–212. 19120297 10.1196/annals.1447.012

[23] Jun H, Yoon Y (2001) The role of viruses in type I diabetes: two distinct cellular and molecular pathogenic mechanisms of virus-induced diabetes in animals. Diabetologia 44: 271–285. 11317656 10.1007/s001250051614

[24] Dogusan Z, Garcia M, Flamez D, Alexopoulou L, Goldman M, et al (2008) Double-stranded RNA induces pancreatic beta-cell apoptosis by activation of the toll-like receptor 3 and interferon regulatory factor 3 pathways. Diabetes 57: 1236–1245. 18223009 10.2337/db07-0844

[25] Eizirik D, Colli M, Ortis F (2009) The role of inflammation in insulitis and beta-cell loss in type 1 diabetes. Nat Rev Endocrinol 5: 219–226. 19352320 10.1038/nrendo.2009.21

[26] Goto M, Eich TM, Felldin M, Foss A, Kallen R, et al (2004) Refinement of the automated method for human islet isolation and presentation of a closed system for in vitro islet culture. Transplantation 78: 1367–1375. 15548977 10.1097/01.tp.0000140882.53773.dc

[27] Oberste MS, Maher K, Kilpatrick DR, Flemister MR, Brown BA, et al (1999) Typing of human enteroviruses by partial sequencing of VP1. J Clin Microbiol 37: 1288–1293. 10203472 10.1128/jcm.37.5.1288-1293.1999 PMC84754

[28] Lennette EH (1969) General principles underlying laboratory diagnosis of viral and rickettsial infections. In: Lennette EH, Schmidt NJ, eds. Diagnostic procedures for viral and rickettsial infections. New York: American Public Health Association 1–63.

[29] Tamura K, Dudley J, Nei M, Kumar S (2007) MEGA4: Molecular Evolutionary Genetics Analysis (MEGA) software version 4.0. Molecular Biology and Evolution 24: 1596–1599. 17488738 10.1093/molbev/msm092

[30] Roivainen M, Ylipaasto P, Savolainen C, Galama J, Hovi T, et al (2002) Functional impairment and killing of human beta cells by enterovirus: The capacity is shared by a wide range of serotypes, but the extend is characteristic of individual virus strains. Diabetologia 45: 693–702. 12107750 10.1007/s00125-002-0805-x

[31] Skog O, Korsgren O, Frisk G (2011) Modulation of innate immunity in human pancreatic islets infected with enterovirus in vitro. J Med Virol 83: 658–664. 21328381 10.1002/jmv.21924

[32] Schulte BM, Kramer M, Ansems M, Lanke KH, van Doremalen N, et al (2010) Phagocytosis of enterovirus-infected pancreatic beta-cells triggers innate immune responses in human dendritic cells. Diabetes 59: 1182–1191. 20071599 10.2337/db09-1071 PMC2857898

[33] Szopa T, Ward T, Taylor K (1992) Disturbance of mouse pancreatic beta-cell function following echo 4 virus infection. Biochem Soc Trans 20.315S. 10.1042/bst020315s 1486985

[34] Uriarte A, Molina G, Cabrera-Rode E, Ventura R, Vargas J, et al (1991) Prospective study in children with high risk of type I diabetes as a sequel of Echo-4 virus infection: 1986–1989. Rev Cub Endocrinol 2: 34–43.

[35] Elshebani A, Olsson A, Westman J, Tuvemo T, Korsgren O, et al (2007) Effects on isolated human pancreatic islet cells after infection with strains of enterovirus isolated at clinical presentation of type 1 diabetes. Virus Res 124: 193–203. 17169456 10.1016/j.virusres.2006.11.004

[36] Horwitz MS, Bradley LM, Harbertson J, Krahl T, Lee J, et al (1998) Diabetes induced by Coxsackie virus: initiation by bystander damage and not molecular mimicry. Nat Med 4: 781–785. 9662368 10.1038/nm0798-781

[37] Richer M, Horwitz M (2009) Coxsackievirus infection as an environmental factor in the etiology of type 1 diabetes. Autoimmunity Rev 8: 611–615. 19393207 10.1016/j.autrev.2009.02.006

[38] Ylipaasto P, Kutlu B, Rasilainen S, Rasschaert J, Salmela K, et al (2005) Global profiling of coxsackievirus- and cytokine-induced gene expression in human pancreatic islets. Diabetologia 48: 1510–1522. 15991020 10.1007/s00125-005-1839-7

[39] Nair S, Leung KC, Rawlinson WD, Naing Z, Craig ME (2010) Enterovirus infection induces cytokine and chemokine expression in insulin-producing cells. J Med Virol 11: 1950–1957. 10.1002/jmv.21900 20872723

[40] Ylipaasto P, Smura T, Gopalacharyulu P, Paananen A, Seppänen-Laakso T, et al (2012) Enterovirus-induced gene expression profile is critical for human pancreatic islet destruction. Diabetologia 55: 3273–3283. 22983635 10.1007/s00125-012-2713-z

[41] Blanco P, Palucka AK, Pascual V, Banchereau J (2008) Dendritic cells and cytokines in human inflammatory and autoimmune diseases. Cytokine Growth Factor Rev 19: 41–52. 18258476 10.1016/j.cytogfr.2007.10.004 PMC2413068

[42] Von Herrath M (2009) Can we learn from viruses how to prevent type 1 diabetes?: the role of viral infections in the pathogenesis of type 1 diabetes and the development of novel combination therapies. Diabetes 58: 2–11. 19114721 10.2337/db08-9027 PMC2606872

[43] Al-Hello H, Ylipaasto P, Smura T, Rieder E, Hovi T, et al (2009) Amino acids of Coxsackie B5 virus are critical for infection of the murine insulinoma cell line, MIN-6. J Med Virol 81: 296–304. 19107967 10.1002/jmv.21391

[44] Paananen A, Savolainen-Kopra C, Kaijalainen S, Vaarala O, Hovi T, et al (2007) Genetic and phenotypic diversity of echovirus 30 strains and pathogenesis of type 1 diabetes. J Med Virol 79: 945–955. 17516516 10.1002/jmv.20922

[45] Karvonen M, Viik-Kajander M, Moltchanova E, Libman I, LaPorte R, et al (2000) Incidence of childhood type 1 diabetes worldwide. Diabetes Care 23: 1516–1526. 11023146 10.2337/diacare.23.10.1516

[46] Sarmiento L, Cabrera-Rode E, Lekuleni L, Cubas I, Molina G, et al (2007) Occurrence of enterovirus RNA in serum of children with newly diagnosed type 1 diabetes and islet cell autoantibody-positive subjects in a population with a low incidence of type 1 diabetes. Autoimmunity 40: 540–545. 17966045 10.1080/08916930701523429

[47] Strachan DP (1989) Hay fever, hygiene, and household size. BMJ 299: 1259–1260. 2513902 10.1136/bmj.299.6710.1259 PMC1838109

[48] Cabrera-Rode E, Díaz-Horta O, Toniolo A, Sarmiento L (2013) Type 1 Diabetes in the tropics: A link with enterovirus infections. In: Taylor K, Hyöty H, Toniolo A, Zuckerman A (eds) Diabetes and Viruses. Springer New York, 195–205.

