# Supplementary material for: Expression of Innate Immunity Genes and Damage of Primary Human Pancreatic Islets by Epidemic Strains of Echovirus: Implication for Post-Virus Islet Autoimmunity
Source: PLoS One. 2013 Nov 1;8(11):e77850. doi: 10.1371/journal.pone.0077850 (PMC3815302; doi:10.1371/journal.pone.0077850)
Supplement: Table S1 — Donor information. DI, Dynamic index: n.a, not available. (DOC) [file pone.0077850.s001.doc]

| **Table 1S.** Donor Information | | | | | | |
| --- | --- | --- | --- | --- | --- | --- |
|  |  |  |  |  |  |  |
| Donor | Gender | DI | HLA  A1/A2 | HLA  B1/B2 | HLA  DR1/DR2 | HLA DQ1/DQ2 |
| H1624 | Female | n.a | 1/10 | 8/16 | 3/11 | 2/7 |
| H1625 | Male | 2.2 | 2/n.a | 18/51 | 4/13 | n.a |
| H1627 | Male | 3.4 | 2/n.a | 7/40 | 4/13 | n.a |
| H1631 | Male | 5.8 | 3/n.a | 35/40 | 1/14 | n.a |
| H1632 | Male | 0.8 | 19/28 | 12/27 | 1/7 | n.a |
| H1658 | Male | 9.5 | 1/2 | 35/12 | 4/8 | n.a |
| H1659 | Male | 3.5 | 1/2 | 27/48 | 8/12 | n.a |
| DI, Dynamic index; n.a, not available | | | | | | |
